# Familien mit Säuglingen und Kleinkindern in der COVID-19-Pandemie: Belastungserleben und Entwicklungsauffälligkeiten unter Berücksichtigung der sozialen Lage

**DOI:** 10.1007/s00103-023-03744-x

**Published:** 2023-07-20

**Authors:** Ilona Renner, Susanne M. Ulrich, Anna Neumann, Digo Chakraverty

**Affiliations:** 1 Nationales Zentrum Frühe Hilfen, In der Bundeszentrale für gesundheitliche Aufklärung, Maarweg 149–161, 50825 Köln, Deutschland; 2 Nationales Zentrum Frühe Hilfen, Deutsches Jugendinstitut, München, Deutschland

**Keywords:** Frühe Hilfen, Pandemie, Repräsentativerhebung, Kinderärzte, Familien, Kindergesundheit, Early childhood intervention, Pandemic, Representative survey, Pediatricians, Families, Children health

## Abstract

**Einleitung:**

Inzwischen ist empirisch belegt, dass Familien mit Kindern, die in der COVID-19-Pandemie Bildungs- und Betreuungseinrichtungen nicht besuchen konnten, Nachteile erlebten. Dies gilt insbesondere für Familien in Armutslagen. Über die Situation von Familien mit sehr jungen Kindern ist noch wenig bekannt. Es wird untersucht, inwieweit 1) Familien mit Säuglingen und Kleinkindern ihre Situation in der Pandemie als belastend erlebten und 2) ob es dabei Unterschiede nach der sozialen Lage gab, 3) wie sich die Pandemie aus Eltern- und Ärztesicht auf die Entwicklung der Kinder ausgewirkt hat und 4) inwieweit die soziale Lage auch bei pandemiebedingten Entwicklungsauffälligkeiten eine Rolle spielte.

**Methoden:**

Das Nationale Zentrum Frühe Hilfen hat von April bis Dezember 2022 eine bundesweit repräsentative Erhebung bei Familien mit Kindern von 0 bis 3 Jahren durchgeführt, „Kinder in Deutschland 0‑3 2022“ (KiD 0‑3; *N* = 7818). Die Studie kombinierte eine Elternbefragung zu Belastungen und Ressourcen mit einer kinderärztlichen Dokumentation der kindlichen Entwicklung.

**Ergebnisse:**

Auch Eltern mit sehr jungen Kindern haben ihre Situation in der COVID-19-Pandemie als belastend erlebt. Dabei zeigten sich deutliche Unterschiede je nach sozialer Lage. Sowohl aus Sicht der Eltern als auch im pädiatrischen Urteil hat sich die Pandemie bereits bei Kleinkindern negativ auf die soziale und affektive Entwicklung ausgewirkt. Diese Effekte sind bei Kindern aus armutsbelasteten Familien stärker ausgeprägt als bei Kindern aus Familien ohne Bezug staatlicher Leistungen zur Grundsicherung.

**Diskussion:**

Um die pandemiebedingten psychosozialen Belastungen der Familien abzumildern und die Chancen der Kinder auf ein gesundes, entwicklungsförderliches Aufwachsen zu verbessern, ist es notwendig, Familien in Bedarfslagen stigmatisierungsfrei und niedrigschwellig zu unterstützen.

**Zusatzmaterial online:**

Zusätzliche Informationen sind in der Online-Version dieses Artikels (10.1007/s00103-023-03744-x) enthalten.

## Abstract (en)

**Introduction:**

It is now well established empirically that families and children who could not attend educational and childcare institutions during the COVID-19 pandemic experienced disadvantages. This is particularly true for families in poverty. However, little is known about the situation of families with young children. The aim of the paper is to investigate (1) to what extent families with infants and toddlers also experienced their situation during the pandemic as stressful, (2) whether there were differences depending on social class, (3) how the pandemic affected the healthy development of infants and toddlers, and (4) to what extent class-related differences can also be identified in this.

**Methods:**

The German National Centre for Early Prevention conducted a nationally representative survey of families with children of age 0 to 3 years in April to December 2022, “Kinder in Deutschland 0‑3 2022” (*N* = 7821). The KiD 0‑3 study combines a parent survey on family psychosocial burden and resources with pediatric documentation of child development.

**Results:**

Parents with very young children experienced their situation in the COVID-19 pandemic as stressful. A clear difference depending on social class emerged. Both from the parents’ perspective and in pediatric judgment, the pandemic had a negative impact on social and affective development even in young children. These effects were more pronounced in children from families experiencing poverty than in children from families not receiving basic government benefits.

**Discussion:**

In order to mitigate the psychosocial consequences of the COVID-19 pandemic for families and to improve children’s chances of growing up in a healthy way that promotes their development, it is necessary to support families in need without stigmatization.

## Einleitung

Die Jahre 2020 bis 2022 waren durch die COVID-19-Pandemie geprägt. Familien gehörten in der Rückschau zu den Bevölkerungsgruppen, die von den Maßnahmen zur Eindämmung des Infektionsgeschehens besonders betroffen waren. Diskutiert wurde dies anfangs vor allem mit Blick auf Familien mit Kindern, deren Zugang zu Bildungs- und Betreuungseinrichtungen zum Zeitpunkt der „Lockdowns“ und weiterer Maßnahmen eingeschränkt war [1–4].

Später trat zunehmend ins Bewusstsein der Öffentlichkeit, dass die Pandemie auch frühere Familienphasen überschattete: Ängste und eingeschränkte Kontaktmöglichkeiten in der Schwangerschaft, die Situation im Kreißsaal und die soziale Isolation mit dem Säugling wurden als belastend, teilweise traumatisierend beschrieben [5]. Da gerade die sehr frühe Kindheit, die maßgeblich durch emotionale Beziehungen im familiären Nahbereich geprägt ist, das Fundament für die Persönlichkeitsentwicklung legt [6], muss die Familiensituation rund um die Geburt im Zusammenhang mit den Verwerfungen infolge der Pandemie verstärkt in den Blick genommen werden. Inzwischen liegt eine Metaanalyse US-amerikanischer Untersuchungen vor, die eine erhöhte Prävalenz von Depressionen und Angststörungen (auch) bei Eltern mit sehr jungen Kindern belegt [7]. Dies wurde für die Situation in einem deutschen Bundesland (Bayern) empirisch bestätigt [8].

Neben erhöhten elterlichen Belastungen wurden vereinzelt jedoch (auch) positive Effekte der Pandemie berichtet, wie bspw. eine „Entschleunigung“ des Alltags von Familien mit jungen Kindern [9]. Noch fehlen empirische Ergebnisse zur Verteilung von negativen und positiven Effekten der Pandemie bezogen auf die Gesamtheit junger Familien in Deutschland.

Dass die Pandemie bzw. die damit verbundenen Einschränkungen sich nicht nur auf Eltern, sondern auch auf die Entwicklung und Gesundheit von Kindern auswirkte, ist inzwischen für ältere Kinder und Jugendliche in Deutschland empirisch gut belegt [10–12]. Unklar ist, inwieweit auch die frühkindliche Entwicklung und Gesundheit beeinflusst wurden. Einerseits wurde eine Zunahme von Regulationsschwierigkeiten und kindlichen Entwicklungsverzögerungen beobachtet [13], andererseits gibt es empirische Hinweise auf positive Auswirkungen der Familiensituation in der Pandemie, bspw. bezüglich stärker prosozialen Verhaltens der Kinder [14].

Sozialwissenschaftliche Studien erheben Aspekte der frühkindlichen Gesundheit und Entwicklung oftmals allein über die Einschätzung durch die Eltern. Um ein vollständigeres Bild zu erhalten, wird der Selbstbericht der Eltern in der vorliegenden Studie durch ein pädiatrisches Urteil im Rahmen der U‑Untersuchungen ergänzt.

Forschungsdesiderate gibt es auch mit Blick auf potenzielle Effekte sozialer Ungleichheit: Bereits zu Beginn der Pandemie wurde von Expertinnen und Experten befürchtet, dass Armut die pandemiebedingten Belastungen für Familien und Kinder verstärkt [15]. Inzwischen gibt es empirische Evidenz, dass dies in Deutschland für Familien mit älteren Kindern zutrifft [9, 16, 17]. Junge Familien in Belastungslagen wurden bisher jedoch nur in wenigen empirischen Studien mit geringer Stichprobengröße gezielt in den Blick genommen [18]. Bis heute fehlt ein systematischer empirischer Vergleich zwischen Familien mit sehr jungen Kindern in unterschiedlich stark ausgeprägten Belastungslagen während der COVID-19-Pandemie.

Gefördert aus Mitteln der Bundesstiftung Frühe Hilfen des Bundesministeriums für Familie, Senioren, Frauen und Jugend (BMFSFJ) hat das Nationale Zentrum Frühe Hilfen (NZFH) 2022 eine bundesweit repräsentative Erhebung „Kinder in Deutschland 0‑3 2022“ (KiD 0‑3) mit 7818 teilnehmenden Familien mit Kindern von 0–3 Jahren durchgeführt. Die Studie KiD 0‑3 2022 kombinierte eine Befragung von Eltern zu Belastungen, Ressourcen und der Nutzung von Unterstützungsangeboten mit einer kinderärztlichen Dokumentation des kindlichen Entwicklungsstandes.

Anhand von Auswertungen dieser bundesweit repräsentativen Familiendaten wollen wir folgende Forschungsfragen beantworten:

1. Inwieweit wurde die COVID-19-Pandemie von Familien mit Säuglingen und Kleinkindern als belastend erlebt?
2. Inwieweit hängt die Bewertung von positiven und negativen Aspekten der Pandemie mit der sozialen Lage einer Familie zusammen?
3. Inwieweit hat sich die Situation in der Pandemie auf die soziale, affektive und körperliche Entwicklung der Säuglinge und Kleinkinder ausgewirkt?
4. Inwieweit kann bei den berichteten Auswirkungen der Pandemie auf die frühkindliche Entwicklung ein Einfluss der Soziallage beobachtet werden?

## Methoden

### Studiendesign

Deutschlandweit wurden im April 2022 Kinder- und Jugendärztinnen bzw. -ärzte aus einer repräsentativen Adressdatei nach einem Zufallsprinzip ausgewählt [19]. Die Kinderärztinnen und -ärzte wurden gebeten, sich mit ihren Praxen als Studienzentren zu beteiligen. Eltern, die mit ihren Kindern zur Früherkennungsuntersuchung (U3 bis U7a) in die Praxen kamen, füllten einen Online-Erhebungsbogen zu Belastungen, Ressourcen sowie zur Nutzung von Unterstützungsangeboten rund um die Geburt aus. Zusätzlich gaben die 258 teilnehmenden Ärztinnen oder Ärzte anhand eines Dokumentationsbogens während der Früherkennungsuntersuchung eine Einschätzung zur Gesundheit und Entwicklung des Kindes ab. Beide Datensätze, die Angaben der Eltern im Online-Erhebungsbogen sowie die Angaben der Ärztinnen und Ärzte im ärztlichen Dokumentationsbogen, wurden im Gesamtdatensatz fallbezogen zusammengeführt. Anonymität wurde für alle Beteiligten an der Studie gewährleistet, Datenschutzvorgaben wurden nachprüfbar eingehalten. Durchgeführt wurde die Erhebung von dem Forschungsinstitut House of Research, Berlin. Der Datensatz wurde vom NZFH auf Basis des Mikrozensus 2021 gewichtet, die Auswertungen erfolgten mit dem Statistikprogramm Stata 17. In die unten dargestellten Analysen sind jeweils nur vollständige Antworten eingeflossen (Missing Listwise). Die Auswertungen umfassten deskriptive Statistiken und Chi-Quadrat-Tests. Für die Darstellung signifikanter Unterschiede wurde ein Grenzwert von *p* < 0,05 herangezogen. Zusätzlich wurden signifikante Gruppenunterschiede mit 95 %-Konfidenzintervallen (KI) bewertet. Das Forschungsvorhaben erhielt ein positives Votum der Ethikkommission des Deutschen Jugendinstituts e. V., München.

### Indikatoren

#### Soziale Lage.

Um die soziale Lage einer Familie *näherungsweise* zu bestimmen, wurde in der vorliegenden Arbeit als Proxy der Armutsindikator „Bezug staatlicher Leistungen zur Grundsicherung“ (Arbeitslosengeld II; Sozialgeld nach SGB II; Sozialhilfe; Bedarfsorientierte Grundsicherung) herangezogen. Dieser Indikator hat den Vorteil, dass die Items im Elternfragebogen verständlich und leicht beantwortbar sind – im Gegensatz zu Fragen bspw. zum Familieneinkommen. Dementsprechend kommt es hier auch kaum zu fehlenden Werten. Dabei betrifft Armut vielfältige Aspekte des Daseins auch abseits wirtschaftlicher Deprivation. Nach Bourdieu (1992) ist Armut sogar der zentrale Indikator für die Soziallage eines Menschen, da allein ökonomisches Kapital in soziales und kulturelles Kapital konvertierbar ist [20].

#### Allgemeines Erleben der COVID-19-Pandemie.

Das allgemeine Erleben der Pandemie wurde mit 2 Items auf einer 5‑Punkt-Skala von „trifft gar nicht zu“ bis „trifft voll und ganz zu“ erhoben: „Die Zeit hat mich persönlich/uns als Familie stark belastet“ und „Ich kann dieser Zeit auch gute Seiten abgewinnen“.

#### Potenzielle spezifische Belastungen.

Die Teilnehmenden gaben für 10 spezifische Belastungen (wie bspw. „wenige Kontakte, Einsamkeit“) an, inwiefern diese auf sie und ihre Familie während der Pandemie zutrafen (4-Punkt-Skala von „trifft gar nicht zu“ bis „trifft voll und ganz zu“).

#### Potenziell positive Aspekte.

Die Teilnehmenden gaben für 8 potenzielle positive Aspekte der Pandemie (wie bspw. „Der Alltag wurde entschleunigt“) an, inwiefern sie auf ihre Familie zutreffen oder zugetroffen haben (4-Punkt-Skala von „trifft gar nicht zu“ bis „trifft voll und ganz zu“). Die Items zu potenziellen spezifischen Belastungen und potenziell positiven Aspekten der Pandemie wurden auf Grundlage quantitativer und qualitativer Ad-hoc-Studien des NZFH [18] selbst entwickelt und in einem Pretest mit der Zielgruppe validiert.

#### Auswirkung der Pandemie auf die kindliche Entwicklung.

Sowohl Eltern als auch Kinderärztinnen und -ärzte wurden nach ihrer Einschätzung möglicher Auswirkungen der Pandemie auf die soziale, affektive und körperliche Entwicklung des jeweils untersuchten Kindes gefragt. Ob sich die Pandemie auf die Entwicklung des Kindes ausgewirkt habe, konnten beide Befragtengruppen jeweils mit „Nein“, „Ja, positiv“, „Ja, negativ“, oder „Weiß nicht“ beantworten (Tab. Z1 im Onlinezusatzmaterial). Der ärztliche Dokumentationsbogen wurde in enger Zusammenarbeit mit dem Berufsverband der Kinder- und Jugendärzte e. V. (BVKJ) entwickelt. Der Elternfragebogen, der ärztliche Dokumentationsbogen und die Implementierung der Studie in den Arztpraxen (Studienzentren) wurden umfassenden Pretests unterzogen.

## Ergebnisse

An der Studie haben 7818 Mütter und Väter, darunter auch Adoptiveltern, teilgenommen, die mit ihren Kindern zur U3–U7a in die Kinderarztpraxis kamen. Von 5591 Eltern liegen neben der Einwilligungserklärung und dem ärztlichen Dokumentationsbogen auch vollständig ausgefüllte Elternfragebögen vor. Von den Eltern waren 91,6 % Mütter. 10,3 % der Eltern gaben an, dass sie selbst oder Mitglieder ihres Haushalts in den vergangenen 12 Monaten staatliche Leistungen zur Grundsicherung erhalten hatten. Im Durchschnitt waren die Kinder 15,2 Monate alt (Standardabweichung 0,16).

### Inwieweit wurde die COVID-19-Pandemie von Familien mit Säuglingen und Kleinkindern als belastend erlebt?

Die Mütter und Väter machten zunächst Angaben zum allgemeinen bisherigen Erleben der Pandemie und wurden anschließend gebeten, Fragen zu konkreten Belastungen, Sorgen und Nöten sowie auch zu positiven Seiten der Pandemie für sich selbst und ihre Familie zu beantworten.

Fast ein Viertel (23,7 %) der Teilnehmenden gab an, dass „die Zeit sie persönlich und/oder ihre Familie bisher ‚stark belastet‘“ habe, mehr als ein Drittel (37,5 %) konnte „dieser Zeit auch gute Seiten abgewinnen“. Diese differenzierte Rückschau auf die Auswirkungen der Pandemie auf die Familiensituation spiegelt sich auch in den Zustimmungswerten zu den konkreten negativen und positiven Aspekten der Pandemie: Weit über die Hälfte der Befragten berichtete von der „Angst vor Ansteckung“ (62,2 %), von „Langeweile, da Freizeitangebote nicht stattfanden“ (62,4 %), von der „Sorge um Nachteile für die Kinder“ (60,1 %), von „fehlenden Unterstützungsangeboten“ für Familien (58 %) und von „wenig Kontakten, Einsamkeit“ (57,8 %). Neben diesen zahlreichen negativen Erfahrungen in der Pandemie stimmte ein etwas geringerer Anteil Eltern (auch) einigen Items zu, die positive Erfahrungen in der Pandemie beschreiben, wie die „Entschleunigung“ des Alltags (58,6 %), die Stärkung des Zusammenhalts in der Familie (59,2 %) und, dass weniger Geld ausgegeben wurde (54,2 %).

### Inwieweit hängt die Bewertung von positiven und negativen Aspekten der Pandemie mit der sozialen Lage einer Familie zusammen?

Die Studie offenbart für Familien mit Säuglingen und Kleinkindern die besondere Bedeutung der sozialen Lage sowohl für das allgemeine Erleben der Pandemie als auch für die Bewertung konkreter potenziell positiver und negativer Aspekte.

Fast ein Drittel (31,4 %) der Familien mit Bezug von Leistungen zur Grundsicherung gab an, dass die Zeit während der Pandemie sie durchweg „stark belastet“ habe, gegenüber etwas mehr als einem Fünftel (22,8 %) in der Vergleichsgruppe. Besonders auffällig sind die Gruppenunterschiede in der Extremkategorie: Unter den armutsbelasteten Familien stimmten mehr als doppelt so viele (16,7 % vs. 7,6 %) „voll und ganz“ der Aussage: „Die Zeit hat mich persönlich/uns als Familie stark belastet“, zu. 32 % der Eltern in armutsbelasteten Familien konnten ihrer Situation in der Pandemie auch in der Rückschau keinerlei positive Seiten abgewinnen. In der Vergleichsgruppe waren dies mit 22,9 % signifikant weniger.

Auch die Auswertung der Angaben zu den konkreten Belastungen (Tab. 1) zeigt, dass Familien in Armutslagen insgesamt stärker von negativen Auswirkungen der Pandemie betroffen waren als die Vergleichsgruppe (Belastungs-Gesamtscore durchschnittlich 5,2 vs. 4,6). Der Gesamtscore zeigt jedoch nicht an, dass – je nach Soziallage – unterschiedliche pandemiebedingte Belastungen stärker zum Tragen kamen, mit teilweise gegenläufiger Tendenz: So gaben Eltern in Armutslagen signifikant häufiger „Existenzängste“, „Konflikte in der Familie“, „Lagerkoller“ und „Langeweile“ an. „Angst vor Ansteckung und Corona-Erkrankungen in der Familie“ wurde dagegen häufiger von Familien ohne Armut angegeben.

**Table 1:** 

	Armut (%)	Keine Armut (%)	*p*-Wert
Existenzängste	52,53	22,70	**<** **0,001**
Lagerkoller	44,20	38,50	**0,001**
Langweile	66,02	61,96	**0,001**
Familiäre Konflikte	33,70	21,76	**<** **0,001**
Keine Unterstützungsangebote	55,43	58,28	0,651
Angst vor Ansteckung	57,43	62,77	**0,007**
Einsamkeit	56,80	58,01	0,939
Sorge um Nachteile für Kinder	65,79	59,47	**0,003**
Doppelbelastung	35,77	38,70	0,892
Wegfall von Unterstützung	39,80	42,71	0,688

Angaben von *N* = 5591 Familien (in Prozent), Daten sind gewichtet mittels einer Design-Gewichtung (Bundesland) und Poststratifizierungsgewichtung (Bildung, Staatsangehörigkeit, alleinerziehend). Die Signifikanz der Ergebnisse wurde anhand des Chi-Quadrat-Tests geprüft. Fett markiert sind signifikante Werte (*p* < 0,05)

Auch mit Blick auf die potenziell positiven Aspekte wurde die Pandemie je nach sozialer Lage unterschiedlich erlebt: Familien mit Bezug staatlicher Leistungen zur Grundsicherung äußerten weniger Zustimmung zu den Items, die positive Effekte der Pandemie beschrieben, als Familien der Vergleichsgruppe.

Deutlich fällt der Unterschied bei dem Item: „Wir haben weniger Geld ausgegeben“, aus: Diesen positiven Effekt haben Familien in Armutslagen seltener angegeben als Familien ohne Armutslage. Ähnlich groß ist der Unterschied bei dem Item: „Der Alltag wurde entschleunigt“, dem deutlich mehr Familien ohne Bezug von Leistungen zur Grundsicherung zustimmten (Tab. 2).

**Table 2:** 

	Armut (%)	Keine Armut (%)	*p*-Wert
Zusammenhalt gestärkt	52,65	60,01	0,067
Tagesstruktur verbessert	34,06	28,66	0,078
Weniger Geldausgaben	41,99	55,65	**<** **0,001**
Weniger Druck	40,79	44,02	0,125
Entschleunigter Alltag	48,29	59,89	**<** **0,001**
Partner hat sich mehr eingebracht	48,89	47,26	0,334
Mehr Zeit für mich	22,48	20,66	0,884
Mehr Zeit als Paar	35,74	38,90	0,206

Angaben von *N* = 5591 Familien (in Prozent), Daten sind gewichtet mittels einer Design-Gewichtung (Bundesland) und Poststratifizierungsgewichtung (Bildung, Staatsangehörigkeit, alleinerziehend). Die Signifikanz der Ergebnisse wurde anhand des Chi-Quadrat-Tests geprüft. Fett markiert sind signifikante Werte (*p* < 0,05)

### Inwieweit hat sich die Pandemie auf die soziale, affektive und körperliche Entwicklung der Säuglinge und Kleinkinder ausgewirkt?

Sowohl Eltern als auch Ärztinnen und Ärzte schätzten unabhängig voneinander ein, inwieweit sich die Pandemie auf die soziale, affektive und körperliche Entwicklung der Kinder ausgewirkt hat.

Da die Entwicklung der Kinder im Alter von 0 bis 3 Jahren schnell voranschreitet und jeweils unterschiedliche Entwicklungsschritte und -aufgaben im Vordergrund stehen, werden die Ergebnisse im Folgenden für Säuglinge (0–11,9 Monate) und Kleinkinder (12–48 Monate) getrennt dargestellt.

Für den überwiegenden Teil der Kinder gaben Eltern (Tab. 3) und Ärztinnen bzw. Ärzte (Tab. 4) an, dass die Pandemie weder eine positive noch eine negative Auswirkung auf die kindliche Entwicklung hatte. (Eltern: je nach Entwicklungsbereich sozial, affektiv, körperlich zwischen 58,38 % und 71,57 %/Ärztinnen und Ärzte: zwischen 84,11 % und 93,35 %). Auffällig ist, dass Eltern durchweg mehr (positive wie negative) Auswirkungen der Pandemie auf die kindliche Entwicklung berichteten als Ärztinnen und Ärzte; die Antwortmuster, bezogen auf die einzelnen Entwicklungsbereiche, sind jedoch ähnlich.

**Table 3:** 

*Körperliche Entwicklung*					
*n* = 5520		Nein (%)	Ja, positiv (%)	Ja, negativ (%)	Weiß nicht (%)
F(2,95, 16283,82) = 23,2689	Säuglinge	70,18	4,29	2,15	23,37
*P* < 0,0001	[95 %-KI]	[68,14; 72,14]	[3,47; 5,30]	[1,53; 3,03]	[21,61; 25,24]
	Kleinkinder	73,57	5,93	5,65	14,85
	[95 %-KI]	[71,61; 75,44]	[4,95; 7,09]	[4,64; 6,86]	[13,42; 16,41]
	Total	71,97	5,16	4,00	18,87
	[95 %-KI]	[70,57; 73,33]	[4,49; 5,91]	[3,37; 4,74]	[17,73; 20,07]
*Soziale Entwicklung*					
*n* = 5507		Nein (%)	Ja, positiv (%)	Ja, negativ (%)	Weiß nicht (%)
F(2,99, 16450,61)	Säuglinge	61,91	5,34	6,15	26,60
*P* < 0,0001	[95 %-KI]	[59,75; 64,03]	[4,38; 6,50]	[5,17; 7,29]	[24,72; 28,57]
	Kleinkinder	55,24	6,11	21,27	17,38
	[95 %-KI]	[53,13; 57,33]	[5,13; 7,27]	[19,57; 23,08]	[15,87; 19,01]
	Total	58,38	5,75	14,14	21,73
	[95 %-KI]	[56,87; 59,88]	[5,04; 6,55]	[13,1; 15,26]	[20,51; 22,99]
*Affektive Entwicklung*					
*n* = 5504		Nein (%)	Ja, positiv (%)	Ja, negativ (%)	Weiß nicht (%)
F(2,99, 16446,42)	Säuglinge	68,01	3,61	3,71	24,67
*P* < 0,0001	[95 %-KI]	[65,92; 70,04]	[2,83; 4,59]	[2,9; 4,74]	[22,84; 26,59]
	Kleinkinder	69,01	3,94	8,40	18,65
	[95 %-KI]	[66,99; 70,96]	[3,2; 4,83]	[7,23; 9,73]	[17,03; 20,39]
	Total	68,54	3,78	6,19	21,49
	[95 %-KI]	[67,09; 69,95]	[3,23; 4,42]	[5,45; 7,03]	[20,26; 22,77]

Angaben von *N* = 5591 Familien (in Prozent), Daten sind gewichtet mittels einer Design-Gewichtung (Bundesland) und Poststratifizierungsgewichtung (Bildung, Staatsangehörigkeit, alleinerziehend)

*F* design-basierter F‑Test, *KI* Konfidenzintervall, *n* Stichprobenumfang

**Table 4:** 

*Körperliche Entwicklung*					
*n* = 5565		Nein (%)	Ja, positiv (%)	Ja, negativ (%)	Weiß nicht (%)
F(2,97, 16528,55)	Säuglinge	93,83	1,30	0,61	4,26
*P* = 0,1065	[95 %-KI]	[92,69; 94,81]	[0,91; 1,84]	[0,37; 1,02]	[3,43; 5,28]
	Kleinkinder	92,90	1,71	1,25	4,14
	[95 %-KI]	[91,71; 93,93]	[1,25; 2,32]	[0,85; 1,83]	[3,34; 5,11]
	Total	93,35	1,51	0,95	4,20
	[95 %-KI]	[92,54; 94,07]	[1,20; 1,91]	[0,70; 1,29]	[3,6; 4,88]
*Soziale Entwicklung*					
*n* = 5564		Nein (%)	Ja, positiv (%)	Ja, negativ (%)	Weiß nicht (%)
F(2,96, 16489,54)	Säuglinge	89,14	1,58	1,58	7,70
*P* < 0,0001	[95 %-KI]	[87,68; 90,45]	[1,16; 2,15]	[1,13; 2,22]	[6,56; 9,01]
	Kleinkinder	79,55	2,14	6,79	11,52
	[95 %-KI]	[77,72; 81,26]	[1,64; 2,80]	[5,74; 8,03]	[10,18; 13,01]
	Total	84,11	1,87	4,31	9,70
	[95 %-KI]	[82,93; 85,23]	[1,53; 2,29]	[3,70; 5,02]	[8,80; 10,68]
*Affektive Entwicklung*					
*n* = 5562		Nein (%)	Ja, positiv (%)	Ja, negativ (%)	Weiß nicht (%)
F(2,98, 16596,84)	Säuglinge	88,89	1,53	1,28	8,31
*P* < 0,0001	[95 %-KI]	[87,44; 90,19]	[1,11; 2,09]	[0,87; 1,87]	[7,16; 9,62]
	Kleinkinder	82,04	1,77	3,78	12,41
	[95 %-KI]	[8,03; 83,65]	[1,29; 2,41]	[3,03; 4,72]	[11,03; 13,93]
	Total	85,30	1,65	2,59	10,45
	[95 %-KI]	[84,17; 86,37]	[1,32; 2,06]	[2,14; 3,14]	[9,53; 11,46]

Angaben von *N* = 5591 Familien (in Prozent), Daten sind gewichtet mittels einer Design-Gewichtung (Bundesland) und Poststratifizierungsgewichtung (Bildung, Staatsangehörigkeit, alleinerziehend)

*F* design-basierter F‑Test, *KI* Konfidenzintervall, *n* Stichprobenumfang

Über alle Entwicklungsbereiche hinweg wurden sowohl von Eltern als auch von Ärztinnen und Ärzten bei Kleinkindern häufiger negative Auswirkungen der Pandemie beobachtet als bei Säuglingen (Tab. 3 und 4). Dabei unterschied sich die Häufigkeit der Entwicklungsauffälligkeiten, die der Situation in der Pandemie zugeschrieben wurden, stark nach Entwicklungsbereich. Während die körperliche Entwicklung kaum (negativ) beeinflusst wurde, hatte die Pandemie sowohl aus Perspektive der Eltern als auch der Ärztinnen und Ärzte einen signifikant negativen Einfluss auf die soziale und affektive Entwicklung.

Dies galt in besonderem Maße für die Kleinkinder. Etwa ein Fünftel (21,3 %) der Eltern von 12 bis 48 Monate alten Kleinkindern berichtete von negativen Einflüssen der Pandemie auf die soziale Entwicklung der eigenen Kinder (im Elternfragebogen umschrieben mit „Reaktion auf andere Menschen“). Ärztinnen und Ärzte dokumentierten negative Effekte bei 6,8 % der Kleinkinder. Deutlich seltener wurden negative Auswirkungen der Pandemie auf die soziale Entwicklung von Säuglingen (0–11,9 Monate) angegeben (Eltern: 6,2 %, Ärztinnen und Ärzte: 1,2 %).

Auffällig ist, dass sich sowohl aus Perspektive der Eltern als auch der Ärztinnen und Ärzte die positiven und negativen Auswirkungen der Pandemie auf die kindliche Entwicklung bei Säuglingen die Waage hielten. Mit zunehmendem Alter schienen die negativen Auswirkungen der Pandemie stärker zum Tragen zu kommen: Bei der sozialen und affektiven Entwicklung verdoppelte bzw. verdreifachte sich mit dem Kleinkindalter die Differenz zwischen den Anteilen positiver und negativer Effekte der Pandemie.

### Inwieweit kann bei den berichteten Auswirkungen der Pandemie auf die frühkindliche Entwicklung ein Einfluss der Soziallage beobachtet werden?

Bei der sozialen und affektiven Entwicklung von Kleinkindern im Alter zwischen 12 und 48 Monaten wurden von Eltern sowie Ärztinnen und Ärzten pandemiebedingte negative Effekte beobachtet. Die nach Armutslage aufgeschlüsselten Ergebnisse zeigen, dass negative Auswirkungen der Pandemie auf die *affektive Entwicklung* abhängig von der sozialen Lage der Familie waren: Die jeweiligen Anteile unter Kleinkindern aus armutsbelasteten Familien waren jeweils etwa doppelt so hoch wie unter Kindern aus Familien ohne Bezug staatlicher Leistungen zur Grundsicherung. Dies gilt sowohl für die Auswertungen auf Basis der Elternbefragung (Tab. 5: 16,55 % vs. 7,36 %) als auch auf Basis der ärztlichen Dokumentation (Tab. 6: 6,65 % vs. 3,45 %). Differenzierter stellte sich die Situation hinsichtlich der *sozialen Entwicklung* der Kleinkinder dar: Während Ärztinnen und Ärzte bei armutsbelasteten Familien häufiger negative Effekte der Pandemie auch auf die soziale Entwicklung des Kindes feststellten (Tab. 6), gaben Eltern in Armutslagen im Selbstbericht etwas seltener negative Effekte an als Eltern der Vergleichsgruppe (Tab. 5).

**Table 5:** 

*Soziale Entwicklung*					
*n* = 2873		Nein (%)	Ja, positiv (%)	Ja, negativ (%)	Weiß nicht (%)
F(3,00, 8606,42)	Keine Armut (%)	55,44	5,62	21,68	17,27
*P* = 0,0731	[95 %-KI]	[53,24; 57,61]	[4,64; 6,79]	[19,88; 23,59]	[15,70; 18,95]
	Armut (%)	53,44	10,31	18,41	17,84
	[95 %-KI]	[46,11; 60,64]	[6,68; 15,60]	[13,52; 24,56]	[12,83; 24,25]
	Total (%)	55,22	6,13	21,32	17,33
	[95 %-KI]	[53,11; 57,31]	[5,15; 7,29]	[19,61; 23,13]	[15,81; 18,96]
*Affektive Entwicklung*					
*n* = 2873		Nein (%)	Ja, positiv (%)	Ja, negativ (%)	Weiß nicht (%)
F(2,94, 8438,69)	Keine Armut (%)	69,95	3,73	7,36	18,95
*P* = 0,0001	[95 %-KI]	[67,87; 71,60]	[2,98; 4,66]	[6,25; 08,67]	[17,26; 20,77]
	Armut (%)	61,55	5,77	16,55	16,13
	[95 %-KI]	[54,14; 68,46]	[3,42; 9,56]	[11,65; 22,98]	[11,25; 22,59]
	Total (%)	69,04	3,95	8,37	18,64
	[95 %-KI]	[67,01; 70,99]	[3,22; 4,85]	[7,20; 09,71]	[17,02; 20,38]

Angaben von *N* = 5591 Familien (in Prozent), Daten sind gewichtet mittels einer Design-Gewichtung (Bundesland) und Poststratifizierungsgewichtung (Bildung, Staatsangehörigkeit, alleinerziehend)

*F* design-basierter F‑Test, *KI* Konfidenzintervall, *n* Stichprobenumfang

**Table 6:** 

*Soziale Entwicklung*					
*n* = 2870		Nein (%)	Ja, positiv (%)	Ja, negativ (%)	Weiß nicht (%)
F(2,98, 8536,62)	Keine Armut (%)	80,00	2,22	6,30	11,48
*P* = 0,1543	[95 %-KI]	[78,09; 81,77]	[1,68; 2,93]	[5,24; 7,56]	[10,08; 13,05]
	Armut (%)	76,43	1,57	10,49	11,51
	[95 %-KI]	[69,79; 81,98]	[0,56; 4,37]	[6,77; 15,89]	[7,71; 16,84]
	Total (%)	79,61	2,15	6,76	11,48
	[95 %-KI]	[77,78; 81,32]	[1,64; 2,81]	[5,71; 7,99]	[10,15; 12,97]
*Affektive Entwicklung*					
*n* = 2869		Nein (%)	Ja, positiv (%)	Ja, negativ (%)	Weiß nicht (%)
F(2,74, 7868,51)	Keine Armut (%)	82,24	1,88	3,45	12,42
*P* = 0,0792	[95 %-KI]	[80,42; 83,93]	[1,36; 2,61]	[2,70; 4,40]	[10,98; 14,03]
	Armut (%)	81,00	0,87	6,65	11,47
	[95 %-KI]	[74,8; 85,97]	[0,32; 2,38]	[3,82; 11,32]	[7,67; 16,81]
	Total (%)	82,11	1,77	3,80	12,32
	[95 %-KI]	[80,37; 83,72]	[1,30; 2,42]	[3,04; 4,74]	[10,94; 13,84]

Angaben von *N* = 5591 Familien (in Prozent), Daten sind gewichtet mittels einer Design-Gewichtung (Bundesland) und Poststratifizierungsgewichtung (Bildung, Staatsangehörigkeit, alleinerziehend)

*F* design-basierter F‑Test, *KI* Konfidenzintervall, *n* Stichprobenumfang

## Diskussion

Die bundesweit repräsentative Studie KiD 0‑3 2022 bestätigt, dass auch Eltern mit sehr jungen Kindern die Situation in der COVID 19-Pandemie als stark belastend erlebt haben. Dabei zeigte sich ein deutlicher Zusammenhang mit der Soziallage einer Familie sowohl bei der Intensität des Belastungserlebens im Allgemeinen als auch bei einzelnen negativ und positiv bewerteten Aspekten der Familiensituation in der Pandemie. Darüber hinaus zeigen die Analysen, dass die Pandemie sich sowohl aus Sicht der Eltern als auch im pädiatrischen Urteil bei einem Teil der Kleinkinder (12–48 Monate) negativ auf die soziale und affektive Entwicklung ausgewirkt hat. Es gibt zudem Hinweise darauf, dass auch die negativen Effekte der Pandemie auf die kindliche Entwicklung abhängig von der Soziallage sind: Im pädiatrischen Urteil waren diese Effekte bei Kindern aus armutsbelasteten Familien signifikant stärker ausgeprägt als bei Kindern aus Familien ohne Bezug staatlicher Leistungen zur Grundsicherung.

Dass Eltern mit KiTa- und Schulkindern die Situation in der Pandemie als besonders belastend empfanden und dass die gesunde, altersgerechte Entwicklung vieler Kinder und Jugendlicher aufgrund ihrer Situation in der Pandemie gefährdet ist, ist Ergebnis des Abschlussberichtes der interministeriellen Arbeitsgruppe „Gesundheitliche Auswirkungen auf Kinder und Jugendliche durch Corona“ (08.02.2023; [21]). Mit der Studie KiD 0‑3 2022 konnte gezeigt werden, dass beides auch bei Familien mit sehr jungen Kindern (Altersdurchschnitt 15,2 Monate) beobachtet werden kann. Dabei ist nicht ausgeschlossen, dass die pandemiebedingte Belastung bei einem Teil der Familien auch von zeitweise fehlenden Bildungs- und Betreuungsangeboten für das Kind, auf das sich die Eltern- und Arztangaben beziehen, oder für ältere Geschwisterkinder herrührte.

Hinweise aus qualitativen Studien, dass einige Eltern mit der Pandemie auch positive Veränderungen ihres Alltags verbanden [9], konnten auf Basis der KiD 0‑3 2022 bundesweit repräsentativ quantifiziert werden. Interessant ist, dass sich dabei der häufig beobachtete Zusammenhang mit der Soziallage bei den Pandemiefolgen umso deutlicher offenbarte: Armutsbelastete Familien konnten der Pandemie seltener positive Seiten abgewinnen und waren signifikant stärker von den pandemiebedingten Zusatzbelastungen betroffen als die Vergleichsgruppe ohne Bezug staatlicher Leistungen zur Grundsicherung – und das auch aus anderen Gründen. Sorgen bereiteten speziell diesen Familien vor allem Nachteile für die Kinder, die Existenzsicherung, „Lagerkoller“ und Konflikte in der Familie, was im Einklang mit Ergebnissen verschiedener qualitativer Erhebungen bei Fachkräften der Frühen Hilfen und Müttern in Belastungslagen steht [18]. Die Art der Sorgen, die speziell von armutsbelasteten Eltern häufiger genannt wurden, erscheint insgesamt und im Vergleich als besonders gravierend.

Potenziell positive Effekte der Pandemie auf den Familienalltag wie (mangels Freizeit- und Urlaubsaktivitäten) „weniger Geld ausgeben“ oder eine „Entschleunigung“ des Alltags fielen bei armutsbelasteten Eltern weniger stark ins Gewicht: Es ist zu vermuten, dass Ersteres in Zusammenhang mit einem ohnehin eingeschränkten Budget steht [22].

Die Situation in der Pandemie scheint sich (auch) in der hier untersuchten sehr jungen Altersgruppe von 0–3 auf verschiedene Aspekte der kindlichen Entwicklung ausgewirkt zu haben. Sowohl aus Sicht der Eltern als auch im pädiatrischen Urteil wurden negative Effekte insbesondere mit Blick auf die soziale und affektive Entwicklung von Kleinkindern zwischen 12 und 48 Monaten gesehen. Vertiefende Analysen zeigten, dass sich die Angaben von Eltern und Ärztinnen und Ärzten grundsätzlich nicht widersprachen, das pädiatrische Urteil jedoch insgesamt zurückhaltender ausfiel als die Einschätzung der Eltern: Berichteten bspw. Eltern von negativen Auswirkungen, gaben Ärztinnen und Ärzte häufig an, sie sähen keine Veränderung oder sie machten keine Angabe. Das eher zurückhaltende ärztliche Urteil kann möglicherweise dadurch erklärt werden, dass Ärztinnen und Ärzte aufgrund ihres Erfahrungswissens und der Vergleichsmöglichkeiten vorsichtiger urteilen als Eltern. Dabei darf die potenzielle Validität der in diesem Fall stärker negativ eingefärbten Sichtweise der Eltern nicht unterschätzt werden: In einer Längsschnittanalyse wurde bspw. gezeigt, dass Kinder, die nach Aussage ihrer Eltern eine frühe Entwicklungsauffälligkeit aufweisen, mit signifikant höherer Wahrscheinlichkeit von ihren Eltern 2 Jahre später weiterhin als auffällig beschrieben werden als Kinder ohne frühe Auffälligkeiten [23].

Die Aufschlüsselung dieser Ergebnisse nach Familien mit und ohne Armutsbelastung offenbart auch bei den Auswirkungen der Pandemie auf die kindliche Entwicklung einen Einfluss der Soziallage: Trotz des insgesamt eher zurückhaltenden ärztlichen Urteils wurden bei einem signifikant höheren Anteil von Kleinkindern aus armutsbelasteten Familien in den U‑Untersuchungen negative Effekte der Pandemie auf die soziale und affektive Entwicklung beschrieben als bei Kindern aus nicht-armutsbelasteten Familien.

Im Gegensatz zur ärztlichen Einschätzung gaben Eltern in Armutslagen jedoch *häufiger an, dass* die Pandemie sich *positiv* auf die soziale Entwicklung ihres Kleinkindes ausgewirkt hat. Dieser Befund könnte darauf hinweisen, dass Kinder innerhalb des Familiengefüges versuchen, Elternstress, der gerade bei armutsbelasteten Familien durch die Pandemie noch zusätzlich verstärkt wurde, mit besonders prosozialem Verhalten zu kompensieren, eine Hypothese, die He et al. 2021 vorgeschlagen haben [14]. Die Diskrepanz zwischen Elterneinschätzung und pädiatrischem Urteil könnte aber auch auf eine möglicherweise geringere Validität der eher subjektiven, von emotionaler Nähe zum Kind geprägten und deshalb womöglich verzerrten Angaben von Eltern verweisen. Wobei nicht auszuschließen ist, dass auch ärztliche Urteile von Verzerrungen betroffen sind, etwa aufgrund von (auch) in der deutschen Gesellschaft weit verbreiteten Stereotypen gegenüber armutsbelasteten Menschen [24]. Umso wichtiger, dass in KiD 0‑3 mit Elternbefragung und ärztlicher Dokumentation beide Perspektiven gleichberechtigt eingeflossen sind.

Verschiedene Einschränkungen bei der Interpretation der hier dargestellten Ergebnisse müssen hingenommen werden. So handelt es sich um ein Querschnittsdesign, bei dem keine Aussagen über kausale Wirkketten getroffen werden können. Darüber hinaus basieren die Daten auf Einschätzungen von Eltern und Ärztinnen bzw. Ärzten, die durch verzerrte Erinnerungen verfälscht sein können (Recall Bias). Weiter deckt die Studie nicht den gesamten Zeitraum der COVID-19-Pandemie ab und ist daher nicht für die Erfahrungen während des gesamten Zeitraums aussagekräftig.

## Fazit

In der Schwangerschaft und den ersten Lebensjahren eines Kindes werden die Weichen für ein gesundes Aufwachsen gestellt. Entwicklungsschritte, die das Kind nicht altersgerecht vollziehen kann, könnten die Bewältigung späterer, darauf aufbauender Entwicklungsaufgaben beeinträchtigen, insbesondere dann, wenn nicht adäquat darauf reagiert wird und nicht ausreichend familiäre Ressourcen für eine Förderung vorhanden sind. Aus diesem Grund ist es notwendig, Familien von Beginn an so zu unterstützen, dass es ihnen gelingt, das Umfeld ihrer Kinder entwicklungsförderlich zu gestalten. Die COVID-19-Pandemie wurde von vielen Familien mit sehr jungen Kindern als belastend erlebt. Bereits Säuglinge und insbesondere Kleinkinder zeigten nach elterlicher und ärztlicher Einschätzung Entwicklungsauffälligkeiten – ausgelöst durch die Familiensituation während der Pandemie. Pandemiebedingte Belastungen und negative Coronafolgen erlebten jedoch nicht alle Familien im gleichen Ausmaß: Besonders betroffen waren Familien und Kinder in Armutslagen. Um negative Auswirkungen der Pandemie auf Familien abzumildern und die Chancen der Kinder auf ein gesundes, entwicklungsförderliches Aufwachsen zu verbessern, ist es notwendig, niedrigschwellige, stigmatisierungsarme Angebote vorzuhalten, die gerade auch armutsbelastete Familien gut erreichen können.

## Supplementary Information




