# Supplementary material for: Families with infants and toddlers in the COVID-19 pandemic: parental stress and child development considering social class
Source: Bundesgesundheitsblatt Gesundheitsforschung Gesundheitsschutz. 2023 Jul 20;66(8):911–9. [Article in German] doi: 10.1007/s00103-023-03744-x (PMC10371926; doi:10.1007/s00103-023-03744-x)
Supplement: Supplementary file 1 [file 103_2023_3744_MOESM1_ESM.pdf]

Onlinematerial

Tabelle Z1: Übersicht über die Items zur kindlichen Entwicklung im Elternfragebogen und ärztlichem Dokumentationsbogen in der Studie KiD 0-3 2022

Befragte	Eltern (Online Fragebogen)	Ärztinnen und Ärzte (Dokumentationsbogen)
Einführungstext	Was denken Sie, hat sich die Corona-Pandemie Ihrem Eindruck nach auf die Entwicklung des Kindes, mit dem Sie heute in der Arztpraxis sind, in den folgenden Bereichen ausgewirkt und wenn ja, wie?	Viele Folgen der Corona-Pandemie sind noch nicht bekannt. Uns interessiert Ihr fachlicher Eindruck, auch im Vergleich zu Gleichaltrigen vor der Corona-Pandemie: Hat sich die Pandemie auf die Entwicklung des Kindes ausgewirkt? Bitte schätzen Sie anhand der folgenden Bereiche ein.<br>Folgende Bereiche der Entwicklung des Kindes wurden beeinflusst:
Körperliche Entwicklung	Körperliche Entwicklung (Ihr Kind hat z.B. mehr als normal zu- oder abgenommen; es hat sich mehr oder weniger bewegt als sonst)	Die körperliche Entwicklung
Antwortkategorien	Nein / ja, positiv / Ja, negativ / Weiß nicht	
Soziale Entwicklung	Reaktion auf andere Menschen (Ihr Kind ist z.B. aufgeschlossener geworden oder es hat sich mehr zurückgezogen oder eine größere Angst vor Fremden entwickelt)	Die soziale Entwicklung
Antwortkategorien	Nein / Ja, positiv / Ja, negativ / Weiß nicht	
Affektive Entwicklung	Stimmung (Ihr Kind war oder ist z.B. häufiger gereizt oder es war/ist insgesamt gelassener geworden)	Die Stimmung/der Affekt
Antwortkategorien	Nein / Ja, positiv / Ja, negativ / Weiß nicht	
